# Acute Left Main Coronary Embolization Following Transcatheter Aortic Valve Implantation

**DOI:** 10.7759/cureus.57748

**Published:** 2024-04-07

**Authors:** Yutaro Miyoshi, Natsuhiko Ehara, Toshiaki Toyota, Kite Kim, Yutaka Furukawa

**Affiliations:** 1 Department of Cardiovascular Medicine, Kyoto University Graduate School of Medicine, Kyoto, JPN; 2 Department of Cardiovascular Medicine, Kobe City Medical Center General Hospital, Kobe, JPN

**Keywords:** coronary artery occlusion, percutaneous coronary intervention (pci), myocardial infarction, transcatheter aortic valve replacement (tavr), transcatheter aortic valve implantation (tavi)

## Abstract

Acute coronary occlusion just after transcatheter aortic valve implantation (TAVI) is a rare but fatal complication, with an incidence of less than 1% but a 30-day mortality rate of up to 50%. The most likely mechanism of acute coronary occlusion following TAVI is the obstruction by the native aortic valve leaflet. However, acute coronary occlusion due to embolus has been rarely reported, and we herein report the case. An 80-year-old woman with severe aortic stenosis and chronic myelogenous leukemia (CML) underwent transfemoral TAVI with a 23-mm balloon-expandable valve. Just before leaving an operation room about 30 minutes after the TAVI procedure, she went into cardiopulmonary arrest. Emergent coronary angiography showed the occlusion of the middle to the distal left main coronary artery with a large embolus. Percutaneous coronary intervention (PCI) was immediately performed, and a drug-eluting stent was eventually placed to improve good coronary flow. She was finally discharged on foot without any other complications and was doing well one year after TAVI with normal left ventricular systolic function and no in-stent restenosis. Considering the transthoracic echocardiography before TAVI and the intravascular ultrasound findings during PCI, it was most likely thought to be caused by the embolus of the degenerated aortic valve tissue. In conclusion, although acute coronary occlusion by embolization following the TAVI procedure is exceedingly rare, we could successfully rescue the patient with immediate PCI.

## Introduction

Aortic stenosis (AS) is often caused by aging-related degeneration of the aortic valve. The prognosis for AS patients with symptoms such as heart failure, syncope, or chest pain is particularly poor [1]. Transcatheter aortic valve implantation (TAVI) is a procedure in which an artificial heart valve is implanted without opening the patient’s chest by using a catheter. TAVI has become an established treatment for patients with symptomatic severe AS, especially in patients at high risk for surgery [2]. Although TAVI is a less invasive therapeutic modality compared to surgery, the 30-day all-cause mortality is 2-4%, some of which are associated with serious complications, including aortic rupture or dissection, cardiac tamponade, stroke, permanent pacemaker implantation, and acute coronary obstruction [3-6]. The incidence of acute coronary occlusion following TAVI is low at less than 1% of native valve interventions. However, the prognosis is quite poor once it occurs, with a 30-day mortality rate of approximately 50% and immediate percutaneous coronary intervention (PCI) is crucial to save the patient’s life [7,8].

Here, we present a case of acute left main coronary embolization just after the TAVI procedure and discuss its cause based on the findings during TAVI and PCI. She was successfully rescued by immediate PCI.

## Case presentation

**Figure 1:**
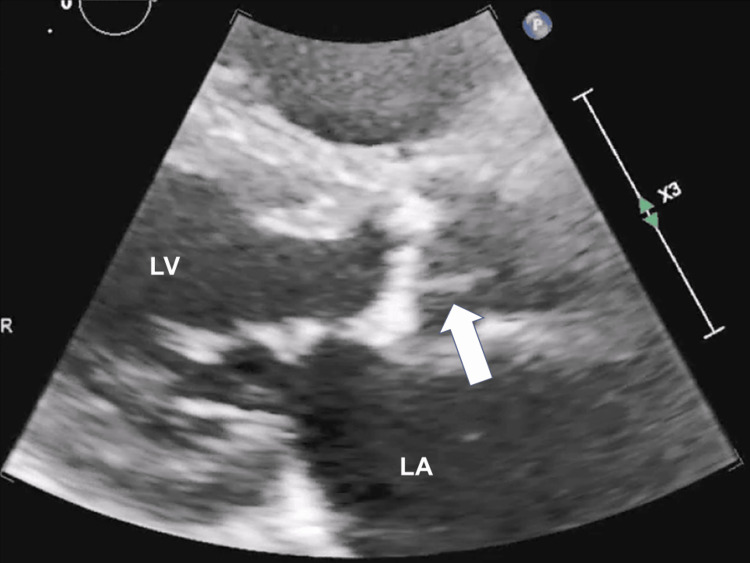
A small mobile structure attached to the aortic valve (arrow) in TTE before TAVI TTE: transthoracic echocardiography, TAVI: transcatheter aortic valve implantation, LA: left atrium, LV: left ventricle

**Figure 2:**
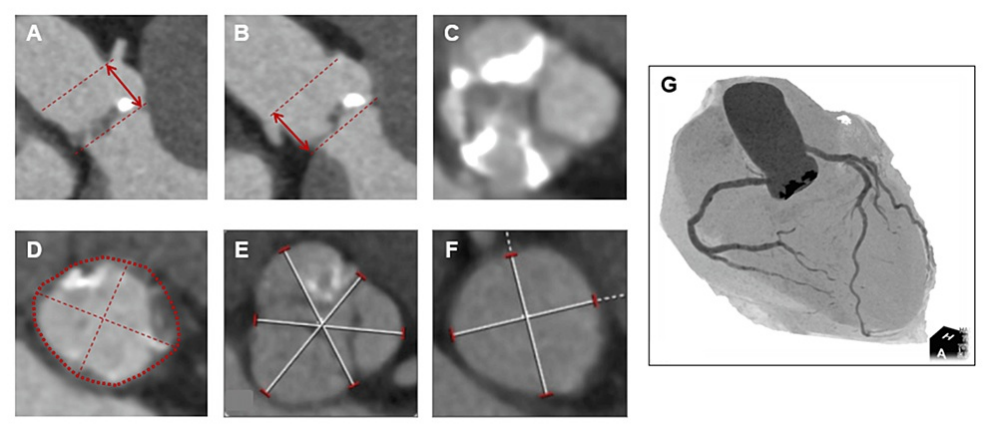
Computed tomography images before TAVI (A) Right coronary artery ostium height: 14.3 mm. (B) Left coronary artery ostium height: 14.8 mm. (C) Severe calcifications on the right and non-coronary cusps. (D) Aortic annulus area: 338 mm^2^, perimeter: 66.1 mm, and diameter: 18.3 mm × 22.1 mm. (E) The sinus of Valsalva: 28.0 mm × 28.5 mm × 28.3 mm. (F) The sino-tubular junction: 24.7 mm×25.3 mm. (G) Angiographic view. TAVI: transcatheter aortic valve implantation

An 80-year-old female patient was referred to our institution for progressive dyspnea on exertion with New York Heart Association (NYHA) Class III symptoms. She had a history of hypertension and dyslipidemia. She also had suffered from chronic myelogenous leukemia (CML) and had been in complete remission for years with dasatinib 20 mg/day. A grade 3 ejection systolic murmur was heard in the left sternal border. The blood chemistry findings revealed anemia (hemoglobin 10.7 g/dl), mild renal dysfunction (creatinine 1.09 mg/dl), normal white blood cell count (6.9×10^3^/μl), normal C-reactive protein (0.07 mg/dl), and elevated N-terminal pro-Brain Natriuretic Peptide (987 pg/ml). Other blood test results showed no remarkable findings (Table 1). Transthoracic echocardiography (TTE) showed severe AS with a peak transvalvular jet velocity of 5.0 m/s, a mean pressure gradient of 52.1 mmHg, an aortic valve area of 0.79 cm^2^, and normal left ventricular ejection fraction of 65.8% with trivial aortic regurgitation. TTE images also demonstrated a small mobile structure attached to the aortic valve (Figure 1, Video 1). Multi-detector computed tomography (CT) measurements showed that the area of the native aortic annulus was 338 mm^2^, the height of the left coronary artery was 14.8 mm, and the diameter of the sinus of Valsalva was 28.0-28.5 mm (Figures 2A-2F). CT coronary angiography demonstrated no significant atherosclerotic plaque (Figure 2G).

**Table 1:** Results of blood cell count and biochemistry

Blood test	Result	Reference range
White blood cells	6,900	3,300-8,600/µl
Hemoglobin	10.7	11.6-14.8 g/dl
Platelet count	155,000	150,000-450,000/µl
C-reactive protein	0.07	< 0.3 mg/dl
Aspartate transaminase	34	13-30 U/l
Alanine transaminase	26	7-23 U/l
Creatine kinase	276	45-250 mg/dl
Blood urea nitrogen	20.9	8-20 mg/dl
Creatinine	1.09	0.46-0.79 mg/dl
Sodium	141	138-145 mmol/l
Potassium	3.7	3.6-4.8 mmol/l
N-terminal pro-Brain Natriuretic Peptide	987	< 125 pg/ml

**Video 1:** TTE image before TAVI TTE: transthoracic echocardiography, TAVI: transcatheter aortic valve implantation

The Society of Thoracic Surgeons (STS) risk score was 5.24%, an intermediate risk. Considering her age, comorbidities, including CML under treatment, and the risk of surgery, transfemoral TAVI under intravenous anesthesia was performed. Activated clotting time was maintained above 250 seconds during the procedure. A 23-mm SAPIEN 3 valve (Edwards Lifesciences, Irvine, CA, USA) was introduced through the right common femoral artery. After confirming the position in aortography, the valve was deployed with -1 ml of underfilling in the planned position. Final aortography showed trivial aortic regurgitation and no obstruction was observed in the left and right coronary arteries. Although sinus bradycardia was prolonged, we finished the procedure.

**Figure 3:**
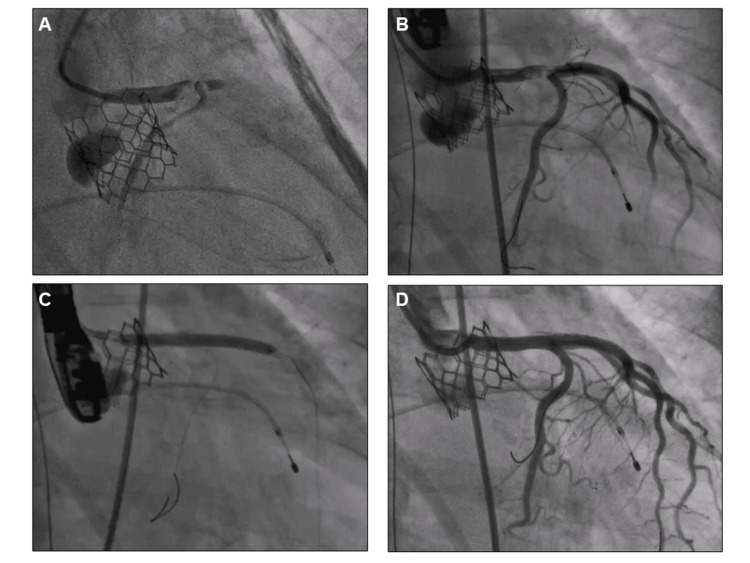
Coronary angiography (A) Total occlusion of the middle to distal left main coronary artery. (B) After balloon dilatation, a filling defect was observed in the distal left main coronary artery. (C) A drug-eluting stent (Ultimaster Tansei 3.5-38 mm, Terumo, Tokyo, Japan) was deployed from the left main to the left anterior descending artery. (D) Final angiography.

**Figure 4:**
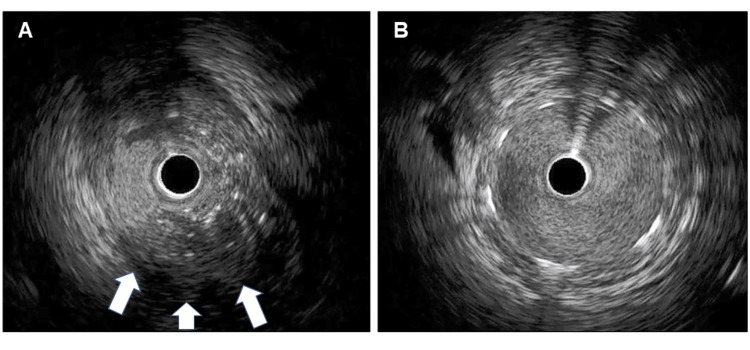
Intravascular ultrasound images (A) A low echoic mass including calcified granules (arrows). (B) A well-dilated drug-eluting stent without protrusion.

Just before leaving the operating room (53 minutes after the implantation of the transcatheter heart valve and 31 minutes later after the end of the TAVI procedure), she complained of chest discomfort and went into cardiopulmonary arrest. Cardiopulmonary resuscitation was started and veno-arterial extracorporeal membrane oxygenation (VA ECMO) was introduced soon after. Transesophageal echocardiography (TEE) showed severely reduced left ventricular function and no pericardial effusion was observed. Coronary angiography showed an abrupt occlusion of the middle to distal left main coronary artery (LMCA) caused by a large embolus (Figure 3A, Video 2), percutaneous coronary intervention was immediately performed. Using a 7 Fr. Judkins Left 3.5 guiding catheter, a 0.014-inch guidewire was crossed from the LMCA to the left anterior descending artery (LAD). First, the LMCA was immediately dilated using several types of balloon catheters, including a 2.5-mm semi-compliant, a 3.5-mm cutting, and a 4.0-mm non-compliant balloon. After balloon dilatation, a filling defect was observed in the distal LMCA and coronary flow was still slow (Figure 3B). Heparin-induced thrombocytopenia (HIT) could not be ruled out and argatroban was administered, but the situation didn’t change (the HIT antibody was found to be negative later). Although manual vacuum aspiration was performed several times using an aspiration catheter, no emboli or thrombi were retrieved at all. Intravascular ultrasound (IVUS) findings detected a low echoic mass, including calcified granules from the LMCA to the mid-LAD (Figure 4A, Video 3). Finally, a drug-eluting stent (Ultimaster Tansei 3.5-38 mm, Terumo, Tokyo, Japan) was deployed from the LMCA to the LAD to get good coronary flow (Figure 3C). Following the stent implantation, coronary angiography showed good coronary blood flow (Figure 3D) and IVUS examination showed good stent apposition without any protrusion (Figure 4B). Intra-aortic balloon pumping (IABP) was also instituted to reduce afterload and maintain coronary blood flow, and the patient was transferred to the intensive care unit.

**Video 2:** Coronary angiography showing the total occlusion of the middle to distal left main coronary artery

**Video 3:** IVUS image indicating a low echoic mass including calcified granules IVUS: intravascular ultrasound

After the procedure, dual antiplatelet therapy (aspirin 100 mg/day and clopidogrel 75 mg/day) was initiated. As cardiac function recovered after the procedure, VA ECMO and IABP were discontinued on day 2 and day 3, respectively. Although peak creatine kinase increased to 3100 IU/L, TTE on day 14 showed an improved ejection fraction of 61.5% and an effective aortic valve area of 1.77 cm^2^. Coronary CT angiography on day 21 demonstrated a well-dilated coronary stent. Brain CT and magnetic resonance imaging showed no obvious findings of cerebral infarction. The postoperative course was quite good and she was discharged on foot on day 35 without any other complications. TTE and CT angiography performed one year after TAVI showed normal aortic valve function, normal left ventricular systolic function, and no in-stent restenosis.

## Discussion

Periprocedural myocardial infarction caused by acute coronary occlusion after TAVI is a rare but fatal complication. Coronary ostia obstruction is typically caused by the displacement of the native aortic valve leaflets and is the main cause of acute coronary occlusion, associated with 0.6%-1.1% of cases [9-11]. PCI is often the treatment of choice for coronary ostia obstruction, with a success rate of 78%-91% [9,11]. Although this was not a problem in our case, low coronary ostia height and small sinus of Valsalva are representative risk factors of coronary ostia obstruction and should be carefully assessed on CT images. On the other hand, periprocedural myocardial infarction due to embolus or thrombus has been rarely reported. Tsujimura et al. described a case of distal coronary embolization during TAVI with histopathological confirmation of the aspirated thrombus compatible with a recent one containing platelets, fibrin, erythrocytes, and leucocytes without calcified components [12].

In our case, it was regretful not to be able to aspirate the embolus and provide a pathological specimen. We might have been able to aspirate the embolus if we had used a larger aspiration catheter or pulled it out while applying negative pressure to the guide catheter. Although there was no histopathological confirmation, we speculated the embolus was unlikely to be a recent thrombus. This is first because heparinization during the procedure was adequate and there was no apparent thrombogenic background, such as atrial fibrillation. Next, this is also because if the embolus was a recent thrombus, some fragmentations would have been made after balloon dilatation, and angiographic findings of distal embolization were observed. The fact that nothing was aspirated by the aspiration catheter in spite of a large embolus might also support this speculation. IVUS findings showed a low echoic mass containing calcified granules, which suggested an organized embolus. We presumed that a small mobile structure attached to the aortic valve detected in TTE images before TAVI was degenerative aortic valve tissue. The mobile structure was not observed in TEE just after the TAVI procedure. The structure seen in the non-coronary cusp in TTE might have been detached from the aortic valve and migrated into the left coronary cusp during wire manipulation, ballooning, or valve implantation and eventually embolized the LMCA. When we encounter cases with similar TTE findings in the future, we should consider performing TEE before TAVI or analyzing CT images in more detail. If the morphology of the aortic valve is judged to be a high risk for TAVI in these multiple modalities, surgical aortic valve replacement might need to be considered.

Van Miegham et al. reported the frequency and histopathology of embolic debris captured by an embolic protection filter during TAVI [13]. Fibrin and thrombotic materials and tissue-derived debris were captured in 74% and 63%, respectively. In one-third of the patients, the tissue fragments consisted of degenerative aortic valve tissue characterized by amorphous calcified material and collagenous and proteoglycan matrix with elastic tissue surrounded by endothelial cells, which might be compatible with the IVUS findings in our case. There was a time difference between the implantation of the transcatheter heart valve and the onset of cardiac arrest. It would be reasonable that coronary obstruction should have happened immediately after valve implantation. We assume that the degenerative aortic valve tissue was trapped between the strut of the transcatheter heart valve and the native aortic valve for a while and was detached subsequently.

## Conclusions

Acute coronary occlusion due to embolization immediately after TAVI is an extremely rare and fatal complication that requires urgent intervention. In our case, considering TTE images before TAVI and the findings during PCI, the embolus was thought to have originated from the degenerated aortic valve tissue. When preoperative TTE shows findings similar to our case, it is suggested that it should be useful to perform TEE or analyze CT in more detail to consider the indication for TAVI again. The patient was successfully treated with cardiopulmonary resuscitation, hemodynamic mechanical support, and PCI. Although the complication was quite severe, immediate intervention could save her life. She was recovered very well thereafter and discharged on foot after about one month.
